# Bacterial Foodborne Illness in Malaysia: *Terminalia* spp. as a Potential Resource for Treating Infections and Countering Antibiotic Resistance

**DOI:** 10.21315/mjms2023.30.2.4

**Published:** 2023-04-18

**Authors:** Matthew James Cheesman, Naveen Kumar Hawala Shivashekaregowda, Ian Edwin Cock

**Affiliations:** 1 School of Pharmacy and Medical Sciences, Gold Coast Campus, Griffith University, Queensland, Australia; 2 Centre for Drug Discovery and Molecular Pharmacology (CDDMP), Faculty of Health and Medical Sciences, Taylor’s University, Selangor, Malaysia; 3 School of Environment and Science, Nathan Campus, Griffith University, Australia

**Keywords:** Terminalia, food poisoning, antibiotic resistance, plant extracts, pathogenic

## Abstract

Acute diarrhoea is becoming a major public health problem in Malaysia, with more than 13.5 million cases reported annually. Foodborne bacterial pathogens are a predominant cause of diarrhoea, with infections causing prolonged illness durations and higher patient mortality rates, placing a tremendous burden on the Malaysian economy. Due to increasing incidences of diarrhoea in Malaysia caused by foodborne pathogens and the increasing levels of resistance towards antibiotics from many different classes, new drugs and/or therapies are urgently required. The evidence for plants as new sources of antibiotics has increased dramatically in recent years and there has been a substantial increase in interest in traditional and herbal medicines. Several *Terminalia* spp. are native to Malaysia, with previous research demonstrating that *Terminalia* spp. are rich in therapeutic phytochemicals and possess antibacterial properties. However, limited research has been conducted on the native Malaysian *Terminalia* spp. for their potential as new antibacterial therapies. The current review discusses the types of bacteria, including antibiotic-resistant strains, that cause food poisoning in Malaysia, and reports the phytochemical content and antibacterial properties of eight of these useful plant species. Future directions pertaining to drug discovery pathways are also suggested.

## Introduction

The World Health Organization (WHO) (1) estimates that diarrhoeal disease kills approximately 525,000 children under 5 years of age each year, with 1.7 billion cases occurring annually. Diarrhoea is most often symptomatic of a microbial infection of the intestinal tract, caused by bacteria, viruses or parasites. The illness most commonly arises from food poisoning, resulting in 550 million people falling ill due to foodborne disease, with 230,000 deaths occurring globally every year (2).

Acute diarrhoea is becoming a major public health problem in Malaysia, with more than 13.5 million cases reported annually (3). Of serious concern, approximately 5% of all cases occur in children under 5 years of age (4). This may have impacts on long-term physiological and psychological development in these children, in addition to the acute health issues. Incidences of food poisoning in Malaysia have significantly increased in recent decades (5), particularly since the consumption of fresh and minimally processed vegetables is becoming very popular among Malaysians (6). Foodborne pathogens are a predominant cause of diarrhoea. Diarrheagenic *Escherichia coli* is a common trigger of childhood diarrhoea (7). *Bacillus cereus* releases diarrheagenic toxins in food poisoning cases (8), while Shigellosis is triggered by *Shigella* spp. including *Shigella flexneri* and *Shigella sonnei* (9). *Aeromonas hydrophila* causes diarrhoea, which may be fatal (10), whilst other foodborne bacteria such as *Salmonella* spp. (11) inhabit the lower gut and cause acute diarrhoea. Additionally, *Klebsiella pneumoniae* has been identified as an entero-invasive foodborne pathogen (12). *Campylobacter* spp. cause foodborne bacterial gastroenteritis (13), while *Vibrio parahaemolyticus* is implicated in up to 50% of the foodborne outbreaks in Southeast Asia (14).

### Pathogens Causing Food Poisoning in Malaysia

There are many Malaysian studies that report the detection of foodborne pathogens in foodstuffs, drinking water, stool samples and clinical isolates. For example, *Escherichia coli* was present in the drinking water in multiple villages in Beluran District, Sabah Malaysia and has been estimated to cause one third of all diarrhoea cases in that region (15). A 20-year study of children suffering from diarrhoea at a medical centre in Kuala Lumpur showed that *Salmonella* spp., enteropathogenic *Escherichia coli*, *Shigella* spp., *Campylobacter* spp. and *Aeromonas* spp. were the pathogens most commonly detected in stool samples (16). Notably, concerns of scientists and medical professionals have been greatly exacerbated by the emergence of bacterial multi-drug resistance (MDR), which has rendered many antibiotics ineffective against intestinal bacterial pathogen infections. Malaysia has experienced an enormous increase in the number of reports of clinical isolates showing antibiotic resistance from patients suffering from food poisoning in recent years. The rise in antimicrobial resistance in foodborne pathogens such as *Staphylococcus aureus*, entero-pathogenic *Escherichia coli*, *Klebsiella pneumoniae*, *Salmonella* spp., *Shigella* spp. and *Campylobacter* spp. have detrimental consequences. Infection with these pathogens may cause longer illness durations and greater patient mortality rates, and they place a tremendous burden on the Malaysian economy (17).

*Staphylococcus aureus* is commonly found on the skin and inside the nose, and thus has previously not generally been considered a foodborne pathogen. However, gastrointestinal (GI) illness is caused by the consumption of foods contaminated with toxins produced by the bacterium and are transmitted by food handling. These ‘Staph food poisoning’ cases are now on the rise worldwide. Some examples of this in Malaysia include *Staphylococcus aureus* contamination of ready-to-eat canned fish sold in Malaysian markets (18) and methicillin-resistant *Staphylococcus aureus* (MRSA) found in raw goat milk from farms in Terengganu (19). One study determined that among 10 locations within the Klang Valley area, 95% of food handlers and 50% of cooked food tested positive for *Staphylococcus aureus*, with some samples containing multi-drug resistant strains (20). MRSA has also been detected in shellfish samples from numerous retail markets in Bangi, Kajang and Serdang, Selangor, and showing high levels of resistance towards amikacin and amoxicillin-clavulanate (21). Recently, the Ministry of Health Malaysia reported that the national prevalence rate of MRSA found in clinical isolates ranges from 17%–28% (22, 23), with resistance observed towards gentamicin, erythromycin and rifampin, in addition to *β*-lactams, with ciprofloxacin resistant foodborne bacteria also recently emerging (24). Indeed, the resistance genes that confer tetracycline (*tetM* and *tetK*), erythromycin (*ermA*, *ermC* and *msrA*) and quinolone (*norA* and *mdeA*) resistance have been detected in MRSA clinical isolates obtained from Malaysian hospitals (25–27). This is concerning as *Staphylococcus aureus* is also known to cause antibiotic-associated nosocomial diarrhoea (28).

Several *Salmonella* spp. serovars have been detected in stool samples of asymptomatic food handlers in Ipoh, Shah Alam and Kuala Terengganu, with most of the isolates showing substantial resistance to a number of different antibiotic classes (29). *Salmonella* spp. have also been identified in vegetables and vegetarian burger patties in Malaysian markets (6, 30) and in chicken and beef samples collected from wet markets, supermarkets and butcher shops (31). MDR *Shigella* spp. strains were first identified in stool samples from various parts of Malaysia in 2002, with more than 70% of the *Shigella flexneri* samples being resistant to at least two antibiotics (32). Another *Shigella* spp. serogroup, *Shigella sonnei*, has been studied in patient stool samples from Malaysian hospitals from different regions from 1997–2009, and was found to be endemic in Malaysia (33). This poses significant health risks of salmonellosis and shigellosis food poisoning to Malaysian consumers from both fresh and prepared food.

*Escherichia coli* is the cause of many cases of diarrhoea in Malaysia. Intestinal diarrheagenic *Escherichia coli* is prevalent in beef, milk, chicken, buffalo and vegetables throughout the country, with some strains being resistant to up to 15 different antibiotics (34). Multiplex PCR has revealed highly resistant ESBL (extended spectrum *β*-lactamase) *Escherichia coli* in milk samples acquired from farms in the Malaysian peninsula (35). *Klebsiella pneumonia* has now been linked to food poisoning cases and can be acquired through contaminated food or water (36), with a highly resistant strain of *Klebsiella pneumonia* detected in almost 50% of street food samples obtained from different states in Malaysia in 2007 (37). ESBL (carbapenem-resistant) *Klebsiella pneumonia* is becoming more frequently identified in clinical faecal samples from Malaysian hospitals and contains a high diversity of carbapenem-resistant genes and sequence types (38–40). Clinical strains from a Malaysian tertiary hospital also possessing the colistin (polymyxin) resistance plasmid *mcr-1* have now also been detected (41). This is especially troubling as colistin is frequently used to treat MDR *Klebsiella pneumonia* infections, leaving few potential therapies for these infections.

Other diarrhoea-triggering pathogens have been identified in foods and clinical samples across Malaysia. *Campylobacter* spp. cause bacterial gastroenteritis arising from food poisoning. Tetracycline and ampicillin-resistant strains have been identified in beef (42) and chicken (43) obtained from wet markets and hypermarkets in Selangor, Malaysia, and this includes both the *Campylobacter jejuni* and *Campylobacter coli* species. *Bacillus cereus* has been confirmed by PCR as being present in both freshly harvested and stored rice samples in various locations in Sarawak (44). This pathogen was also detected in ready-to-eat cooked rice from cafeterias, restaurants and food courts located in five different regions of Pulau Pinang (45) using 16S rRNA gene PCR. Sequencing of this bacteria showed a high prevalence of most diarrheal genes and MDR resistance. Additionally, more than half of the isolates were capable of biofilm formation. This is particularly concerning because this phenotype promotes recurrent contamination in food processing lines. Another foodborne pathogen, *Vibrio parahaemolyticus*, is considered particularly dangerous as it can cause bloody diarrhoea. High microbial loads of *Vibrio* spp. have been detected in raw fish samples from wet markets across Malaysia (46). It should also be noted that in some studies, multiple foodborne pathogens were detected in the same food samples collected for analysis. For example, *Escherichia coli*, *Salmonella* spp. and *Staphylococcus* spp. have all been found in beef and chicken samples acquired from food outlets at ten districts in Kelantan during the month of Ramadhan (47).

### One Health Approach to Antibiotic Resistance

The worsening antibiotic resistance phenomenon has undoubtably been impacted by changes in the environment (including farming practices) and the movement of humans, animals and animal-based products globally. Examples of this include resistance to colistin and its dissemination in sewerage (48, 49) with plasmid-mediated co-resistance also occurring for compounds such as quinolones, ampicillin and heavy metals (50, 51). This further exacerbates the need to incorporate phytochemical compounds in treatment (52) in order to improve the likelihood of eradication of MDR bacteria that cause serious infectious diseases.

### Malaysian Terminalia

Due to increasing incidences of diarrhoea in Malaysia, especially for cases that are associated with foodborne pathogens and the increasing levels of bacterial resistance towards antibiotics from many different classes, new drugs and/or therapies are desperately required. The use of traditional plants as sources of new antibacterial agents has received significantly greater attention in recent years. This is due to the development of numerous currently used medicines that originate from plants, such as artemisinin, but also because of their potential in inhibiting the spread of bacteria, including strains that have evolved high levels of antibiotic resistance. Many plants are rich in bioactive phytochemicals, which are of particular clinical value since their biological activities do not generally confer resistance. One genus of plant that grow in Malaysia is *Terminalia* (53). These plants are renowned for their rich phytochemical content and medicinal properties (54). However, many *Terminalia* spp. found in Malaysia are largely unexplored in terms of their potential as sources of antimicrobial compounds and substantially more research is required. Furthermore, there is an increasing number of reports in the literature on synergistic inhibition of bacterial growth when extracts from different plants are combined, or when plant extracts are combined with conventional antibiotics (55). This is a promising area of research, as it provides evidence that compounds within extracts may not only act as antimicrobials, but also as potentiators of antibacterial activity. It also provides pathways to combat antibiotic resistance, as plant-derived extracts appear to be capable of re-purposing antibiotics that are no longer effective against antibiotic resistant pathogens.

Table 1 provides a summary of the phytochemical content of eight different *Terminalia* plant species found in Malaysia and some of the activities of their extracts against GI bacteria, as well as the phytochemicals contained in these extracts. These plants contain a rich variety of different compounds that have been reported to possess antibacterial activities. Included in these are the egallitannins, which have been found to affect the bacterial membrane integrity and respiratory chain (56, 57). Flavones can form complexes with bacterial proteins and the cell wall, and alkaloids and terpenes inhibit cell division and biofilm formation (58, 59), while both flavonoids and sterols can interact with the penicillin binding protein (60). Glycosides can trigger bacterial cell lysis (61) while other phytochemicals such as lutein promote the digestion of the bacterial cell wall via lysozyme accumulation (62).

The most studied *Terminalia* spp. found in Malaysia and its surrounding areas is *Terminalia catappa*, a tree known by the common Malay name *Ketapang* (63). Extracts prepared from this species have shown promising activity by inhibiting the growth of several foodborne bacteria on agar media and in liquid culture. The methanolic, ethanolic and aqueous leaf extracts contain noteworthy phytochemicals, including cardiac glycosides, saponins, steroids, tannins and phenols, and the extracts inhibit *Escherichia coli* and *Staphylococcus aureus* growth (64–69), as well as *Escherichia coli* isolated from stool samples (70). Fractions separated from organic extracts by thin layer chromatography were also capable of inhibiting *Escherichia coli* growth (71). Other diarrhoea-triggering GI bacteria including *Salmonella* and *Shigella* spp., as well as *Bacillus cereus* and *Aeromonas hydrophila*, were inhibited by aqueous leaf extracts on agar and in liquid broth yielded minimum inhibitory concentration (MIC) values as low as 130 μg/mL (72, 73). Leaves from *Terminalia catappa* extracted with petroleum ether, chloroform or ethyl acetate also inhibit agar growth of foodborne *Escherichia coli*, *Bacillus cereus*, *Listeria monocytogenes*, *Staphylococcus aureus* and *Salmonella typhimurium* pathogens (74, 75). Recently, silver nanoparticles that were synthesised using aqueous *Terminalia catappa* leaf extracts inhibited water-borne *Escherichia coli* and *Staphylococcus aureus* on agar (76). Extracts prepared from *Terminalia catappa* fruit peels inhibit *Salmonella* and *Staphylococcus* spp. (77), and *Klebsiella pneumonia* growth is also inhibited by whole-plant methanol extracts (78).

Of particular interest are the bioactivities that *Terminalia catappa* possess against MDR foodborne pathogens. Bark and leaf extracts inhibit ESBL *Escherichia coli* and *Klebsiella* spp. strains, in addition to MRSA (79), with aqueous extracts inhibiting ESBL *Escherichia coli* found in ready-to-eat foods (80). Moreover, synergistic antibacterial activity was found when methanolic leaf extracts were combined with tetracycline against *Bacillus cereus*, *Escherichia coli* and *Listeria monocytogenes* (70). Interestingly, synergistic growth inhibition of *Staphylococcus aureus* and *Listeria monocytogenes* was also reported when combining *Terminalia catappa* extracts with extracts prepared from other plant species (81). This indicates the potential of *Terminalia catappa* in inhibiting the growth of foodborne pathogens, and that enhanced activities can be achieved when combining extracts with those of other plants, or with conventional antibiotics. The *Terminalia catappa* compounds that are responsible for this activity, or for the activity potentiation, are yet to be determined. Preliminary mass spectrometry experiments conducted on *Terminalia catappa* extracts revealed several general compound classes (82), and protein-ligand studies have provided evidence that a phytochemical present in the extracts may function as a bacterial DNA gyrase inhibitor (83). However, no further information is available in the literature on *Terminalia catappa* antimicrobial molecules.

The only other *Terminalia* species growing in Malaysia with reported antimicrobial activities is *Terminalia citrina* (known locally as *Antoi puteh*, or *Jelawai belang rimau*). Early studies showed that tannins isolated from this plant were active against *Staphylococcus aureus*, *Escherichia coli*, MRSA and *Klebsiella pneumoniae*, although the extracts themselves were not tested (84). Methanolic extracts that were further partitioned into ethyl acetate fractions showed activity towards the foodborne pathogens *Bacillus cereus*, *Staphylococcus aureus* and *Escherichia coli*, as well as numerous *Salmonella*, *Shigella* and *Vibrio* strains (85). An earlier study reported that ethanolic *Terminalia citrina* extracts inhibited the growth of ESBL *Escherichia coli*, with the promising finding that the extract synergistically potentiated norfloxacin to an activity far greater than either the extract or norfloxacin alone (86). However, this study does not appear to have been followed up and warrants further investigation. Despite there being several studies on the antibacterial activities of these two *Terminalia* species, there is little standardisation between studies on the concentrations of extracts tested, making it difficult to compare and contrast the findings. Unfortunately, in many cases the extracts were not resuspended in an aqueous solution and instead reconstituted in 100% dimethyl sulfoxide (DMSO), ethanol or methanol, thus confounding the studies due to the antibacterial inhibition that inherent for the solvents that were used to resuspend the dried crude extracts. This apparent oversight has not been addressed in the relevant reports. Furthermore, the amounts of extracts tested in disc diffusion assays were not specified, and/or often used in high concentrations, further complicating the assessment of the bioactivities.

Very little antimicrobial research has been conducted on many of the other *Terminalia* spp. that can be found in Malaysia. There are reports of anti-proliferative effects of *Terminalia calamansanai* (*Jelawai mentalun*) on carcinoma cells (87), as well as reports that the extracts are rich in ellagitannins (88). However, their antibacterial activities are yet to be reported. *Terminalia copelandii* (*Ketapang*) extracts are rich in flavonoids and phenolic compounds (89), *Terminalia foetidissima* (*djelawai, pelawai* or *gelawai*) leaves contain triterpenoids and sterols (90) and *Terminalia microcarpa* (*Jaha, Jaha kebo, Kalumpit*) leaf extracts possess alkaloids, flavonoids, tannins and terpenoids in abundance (91), although antimicrobial studies on these plants have not been conducted. Two additional plants commonly found in Malaysia are *Terminalia phellocarpa* (*Jelawai Mempelam Babi* tree) and *Terminalia subspathulata* (*Jelawai Jaha*). The medicinal properties of these plants are also unreported.

### Future Directions

The reports of antibacterial activity in *Terminalia catappa* and *Terminalia citrina* (as well as numerous similar studies of *Terminalia* spp. in other regions of the world) indicates that this is a fertile area of research. This is bolstered by findings that show synergistic inhibition of bacterial growth is possible when extracts and/or antibiotics are combined, and this treatment modality is effective against both the susceptible foodborne pathogens as well as their MDR counterparts. In addition, the documented phytochemical content of some of these *Terminalia* spp. indicate that these Malaysian plants are a rich source of the phytochemicals possessing antibacterial properties. Therefore, an expansive study is required to investigate these species further using a panel of *Terminalia* spp., many of which have been overlooked or understudied in terms of drug discovery, in an ongoing search for novel antibiotic therapies. Ultimately, this work may reveal novel drug candidates and/or therapies that can be utilised in the treatment of foodborne diarrheagenic bacterial disease. It may thereby alleviate the increasing burden of hospitalisations caused by food poisoning in Malaysia, as well as stemming the progressively worsening levels of antibiotic resistance that are incurred by cases involving GI MDR pathogens.

It is also important to address the inconsistencies between the various studies, as well as the large gap of knowledge pertaining to these eight *Terminalia* species. This may be achieved by standardising extraction and fractionation protocols, using solvents of different polarities to prepare different extracts from different segments of each plant (leaves, roots, stems and fruit, where applicable) and assessing the antimicrobial activities of each extract using standard assays. Foodborne pathogens such as *Escherichia coli*, *Staphylococcus aureus*, *Bacillus cereus*, *Campylobacter jejuni*, *Klebsiella pneumoniae*, *Shigella sonnei*, *Shigella flexneri*, *Salmonella typhimurium* etc. should be included in such studies, as well as common antibiotic-resistant bacterial strains including MRSA, ESBL *Escherichia coli* and ESBL *Klebsiella pneumoniae*. Active extracts showing activity can subsequently be further examined by testing combinations of the different plant extracts, as well as combinations of extracts with conventional antibiotics (e.g. *β*-lactams, polymyxins B and E, tetracyclines, aminoglycosides, quinolones, and macrolides) to determine precise combinations that lead to synergistic inhibition of bacterial growth (55). Researchers then may use bioactivity-driven separation methodologies to isolate and test multiple fractions from each extract and produce mass spectrometry-based metabolite profiles to help identify compounds of interest with antibacterial and antibiotic potentiation activities. Finally, molecular candidates of interest can be re-tested for antibacterial activity and their mechanisms of action explored.

## Conclusion

Malaysian *Terminalia* spp. are a potential source of new antibacterial therapies that can be utilised in the treatment of foodborne diarrheagenic bacterial disease. However, this resource is yet to be fully examined and utilised and substantially more studies are required to validate activities and explore their uses as therapeutics and food preservatives, as well as products to maintain food preparation and storage hygiene standards. Success in this area would alleviate the increasing burden of hospitalisations caused by food poisoning in Malaysia, as well as to stem the progressively worsening levels of antibiotic resistance that are incurred by cases involving GI MDR pathogens.

## Figures and Tables

**Table 1:** Summary of phytochemical contents and activities against foodborne bacteria for eight *Terminalia* plants found in Malaysia

Species name (and common Malay names)	Phytochemical content	Reported mechanisms of action of phytochemicals	Bacteria inhibited by extracts	References
*Terminalia calamansanai* (*Jelawai mentalun*)	Monomeric, dimeric and trimeric ellagitannins	Perturbation of bacterial membrane and inhibition of bacterial respiratory chain	Unknown	56, 57, 88
*Terminalia citrina* (*Antoi puteh, Jelawai belang rimau*)	Alkaloids, carbohydrates, phenolic compounds, tannins, catechins and saponins, terpenes, and Vitamin E	Cell wall and membrane disruption, complexation with proteins, enzyme inhibition, biofilm and cell cycle inhibition	*Staphylococcus aureus*, *Escherichia coli*, ESBL *Escherichia coli*, MRSA and *Klebsiella pneumoniae*	58, 84–86
*Terminalia copelandii* (*Ketapang*)	Flavonoids, phenolic compounds	Cell membrane disruption	Unknown	58, 89
*Terminalia foetidissima* (*Djelawai, pelawai, gelawai*)	Triterpenes and sterols	Inhibition of biofilm formation, interaction with penicillin binding protein	Unknown	59, 60, 90
*Terminalia catappa* (*Jelawai Ketapang*, Tropical almond)	Cardiac glycosides, saponins, steroids, tannins and phenols, flavonoids, tannins, and anthraquinone glycosides	Cell lysis, complexation with proteins, enzyme inhibition, biofilm and cell cycle inhibition	*Bacillus subtilis*, *Staphylococcusaureus*, *Escherichia coli*, ESBL *Escherichia coli*, *Bacillus cereus*, *Enterococcus faecalis*, *Salmonella typhimurium, Enterobacter aerogenes (Klebsiella aerogenes)*, *Enterobacter cloacae*, *Alcaligenes faecalis*, *Listeria monocytogenes*, *Salmonella typhi*, *Shigella dysenteriae*, *Aeromonas hydrophila*, *Klebsiella pneumonia* and MRSA	58, 61, 64–68, 70–76, 78–83
*Terminalia macrocarpa* (*Jaha, Jaha kebo, Kalumpit*)	Alkaloids, flavonoids, steroids/terpenoids, and gallic and cathecic tannins, squalene, lutein and fatty alcohols	Cell wall and membrane disruption, complexation with proteins, enzyme inhibition, biofilm and cell cycle inhibition, lysozyme accumulation	Unknown	58, 62, 90, 91
*Terminalia phellocarpa* (*Jelawai Mempelam Babi* tree)	Unknown		Unknown	53
*Terminalia subspathulata* (*Jelawai Jaha*)	Unknown		Unknown	53
